# Pulse Wave Transit Time Measurements of Cardiac Output in Septic Shock Patients: A Comparison of the Estimated Continuous Cardiac Output System with Transthoracic Echocardiography

**DOI:** 10.1371/journal.pone.0130489

**Published:** 2015-06-30

**Authors:** Marc Feissel, Ludwig Serge Aho, Stefan Georgiev, Romain Tapponnier, Julio Badie, Rémi Bruyère, Jean-Pierre Quenot

**Affiliations:** 1 Service de Réanimation, Maladies Infectieuses, Centre Hospitalier de Belfort-Montbéliard, Belfort, France; 2 Service d’Epidémiologie et d’Hygiène Hospitalière, Centre Hospitalier Universitaire de Dijon, Bocage Central, Dijon, France; 3 Service de Réanimation Médicale, Centre Hospitalier Universitaire de Dijon, Bocage Central, Dijon, France; 4 INSERM Centre de Recherche UMR866, Université de Bourgogne, Dijon, France; 5 INSERM Centre de Recherche UMR1347, Université de Bourgogne, Dijon, France; University of Florida, UNITED STATES

## Abstract

**Background:**

We determined reliability of cardiac output (CO) measured by pulse wave transit time cardiac output system (esCCO system; CO_esCCO_) vs transthoracic echocardiography (CO_TTE_) in mechanically ventilated patients in the early phase of septic shock. A secondary objective was to assess ability of esCCO to detect change in CO after fluid infusion.

**Methods:**

Mechanically ventilated patients admitted to the ICU, aged >18 years, in sinus rhythm, in the early phase of septic shock were prospectively included. We performed fluid infusion of 500ml of crystalloid solution over 20 minutes and recorded CO by EsCCO and TTE immediately before (T0) and 5 minutes after (T1) fluid administration. Patients were divided into 2 groups (responders and non-responders) according to a threshold of 15% increase in CO_TTE_ in response to volume expansion.

**Results:**

In total, 25 patients were included, average 64±15 years, 15 (60%) were men. Average SAPSII and SOFA scores were 55±21.3 and 13±2, respectively. ICU mortality was 36%. Mean cardiac output at T0 was 5.8±1.35 L/min by esCCO and 5.27±1.17 L/min by CO_TTE_. At T1, respective values were 6.63 ± 1.57 L/min for esCCO and 6.10±1.29 L/min for CO_TTE_. Overall, 12 patients were classified as responders, 13 as non-responders by the reference method. A threshold of 11% increase in CO_esCCO_ was found to discriminate responders from non-responders with a sensitivity of 83% (95% CI, 0.52-0.98) and a specificity of 77% (95% CI, 0.46-0.95).

**Conclusion:**

We show strong correlation esCCO and echocardiography for measuring CO, and change in CO after fluid infusion in ICU patients.

## Introduction

Adequate tissue perfusion and oxygen delivery is the primary goal of therapeutic management in patients with circulatory failure. Cardiac output (CO) is a major determinant of oxygen delivery and is a function of the interaction between the cardiac pump and venous return. Volume expansion is key in order to improve CO[1] and reduce mortality[2]. Furthermore, hemodynamic monitoring plays an important role in the management of these patients, since excessive volume loading may exacerbate the commonly associated respiratory failure[3], limit oxygen diffusion to the tissues by interstitial fluid accumulation[4] and contribute to increased mortality[5].

The optimal monitoring system depends on the individual patient, the current or potential condition for which monitoring is required, and the equipment and expertise available on site[6]. Thermodilution has become the gold standard for the measurement of CO[7], but is now less frequently performed due to its invasive nature.

Many technologies have been developed for minimally invasive monitoring of CO, such as the pulse contour method, based on the relationship between arterial blood pressure and stroke volume. Arterial waveform-based CO is also widely used, but requires arterial puncture[8–9].

One commercially available device is a non-invasive continuous cardiac output measurement method (esCCO, Nihon Kohden, Tokyo Japan) that has recently been validated. It is based on pulse contour analysis combined with pulse wave transit time[10–11]. This method requires only the measurement of peripheral pulse oximetry, and non-invasive blood pressure and ECG-monitoring[12].

Echocardiography has been validated for CO measurement in intensive care patients, particularly in septic shock where the causes of circulatory failure are multiple and often overlap[13–14]. However, echocardiography is not amenable to continuous CO monitoring, and also requires a minimum of training for the operator to be proficient.

In this context, the primary aim of this study was to determine the reliability of CO measured by the esCCO system (CO_esCCO_) as compared with CO measured by transthoracic echocardiography (CO_TTE_) in a homogeneous population of mechanically ventilated patients in the early phase of septic shock. A secondary aim was to evaluate the ability of esCCO to detect a change in CO after fluid infusion.

## Methods

### Patient population

We prospectively screened for eligibility all mechanically ventilated patients admitted to the ICU, older than 18 years, in sinus rhythm, and in the early phase of septic shock (defined as persistent hypotension, despite adequate vascular filling of 20 mL/kg, and need for norepinephrine). We only included patients who required additional fluids because of persistent hypotension (systolic blood pressure < 90 mm Hg), oliguria (urine output < 0.5 mL/kg per hour), or metabolic acidosis (base deficit ≥ 5 or lactates > 2.5 mmol/L). Sedation and analgesia were provided by continuous infusion of midazolam and sufentanil titrated for adaptation without spontaneous breathing (Ramsay score of 4–5).

We excluded patients with an increase in filling pressures defined by transmitral Doppler echocardiography criteria (i.e. E/E’ >15; E/EDT > 5.2)[15] as well as patients in whom the quality of transthoracic echocardiography images was insufficient; patients with intra-aortic balloon pump; patients with severe aortic stenosis; and patients undergoing pacing.

Fluid challenge was performed with a view to achieving mean arterial pressure of 65 mm Hg or higher, and maintaining adequate tissue perfusion. All patients were mechanically ventilated in a volume-controlled mode, with a tidal volume of 6–8 mL/kg of predicted body weight.

The institutional review board (Comité de Protection des Personnes Est I, Dijon) approved the protocol, and considered it to constitute routine clinical practice. The need for informed consent was waived, but all patients or their relatives were given clear information about the study, and their non-opposition was obtained.

### Measurements

EsCCO was displayed continuously on a laptop computer, using a Nihon Kohden BSM9101 bedside monitor (Nihon Kohden, Tokyo, Japan), in which the esCCO system program software (esCCOMon, version 01–04) was installed. The pulse wave transit time is the measure of the interval between the ECG R wave and the pulse plethysmograph upstroke[16]. Details of CO calculation have previously been published elsewhere[11].

All transthoracic echocardiography (TTE) measurement was performed using a CX 50 device (Philips Medical Systems, Andover, MA, USA). Measurements were performed by an investigator (MF, JB) who was blinded to the measurements determined by esCCO. The Doppler echocardiography estimated CO (CO_TTE_) was derived from the Doppler estimated Stroke Volume (SV) using the velocity-time integral (VTI) of flow through the left ventricular outflow tract (LVOT), the diameter of the LVOT, and heart rate recorded during the imaging study[14]. The VTI value was averaged over five consecutive measurements. The method of CO calculation has previously been described[17].

### Intervention

In all patients included in the study, we performed fluid infusion of 500 mL of crystalloid solution over a 20-minute period and recorded CO_esCCO_ and CO_TTE_ before (T0) and 5 minutes after (T1) administration of the fluids. Patients were divided into 2 groups, namely, responders and non-responders, according to the increase in CO_TTE_ in response to volume expansion. In accordance with previous studies[18–19], we used an increase of CO_TTE_ of 15% or more after fluid infusion to differentiate responders from non-responders. The ventilatory settings and the rate of administration of vasoactive drugs remained unchanged throughout the intervention.

### Data recorded

We recorded the following variables for all patients: socio-demographic data, Simplified Acute Physiologic Score (SAPS II)[20] (the worst value within 24 hours after admission), Sepsis-related Organ Failure Assessment (SOFA) score[21] (calculated within the first 24 hours after onset of septic shock, with the most abnormal value for each of the 6 organ systems recorded), origin of sepsis, and outcome.

### Statistical analysis

All data are presented as mean ± SD for quantitative variables and number (percentage) for qualitative variables.

Inter-observer variability was calculated from 3 measures by each observer in each of 10 randomly selected patients, and was expressed as the mean percent error (i.e. the difference between the 2 observed values divided by the mean of the 2 observed values). Intra-observer variability was calculated from 3 CO_TTE_ measures by the same observer from the same 10 randomly selected patients,

Agreement between CO_esCCO_ and CO_TTE_ was assessed in three steps:

Firstly, the relationship between the two methods was estimated using simple linear regression. Robust variance estimator and bootstrapped variance estimator were also used.

Secondly, Pearson’s correlation coefficient between CO_esCCO_ and CO_TTE_ was calculated. Agreement was estimated using the 95% limit of-agreement method developed by Bland and Altman[22], in which the difference between X and Y is plotted against their means, with the 95% confidence intervals. The direction and magnitude of the spread of values around the zero line provides an approximation of any potential systematic bias and random error, respectively. Heteroscedasticity can also be detected where the difference is found to be a function of the mean.

Finally, we computed Lin’s concordance coefficient correlation ρc [23]. Lin’s coefficient combines measures of precision and accuracy to determine whether the observed data deviate significantly from the line of perfect concordance (i.e. the 45° line through the origin). No adjustment for location was performed, since the influence of this variable was not statistically significant.

The limits of agreement for the replicate measurements (pooling of before and after measures) were assessed using a mixed model (estimation of the variance components) as described by Carstensen et al [24].

The diagnostic accuracy of CO_esCCO_ change was expressed as the area under the corresponding receiver operating characteristic curve (ROC). The optimal threshold value was then selected using this ROC curve. Sensitivity (Se), specificity (Sp) and likelihood ratios (LR) were estimated. Confidence intervals of proportions (Se, Sp) were assessed using the binomial exact method.

All statistical analyses were performed using STATA software version 12.0 (Statacorp, College Station, TX, USA).

## Results

### Study population

From a total of 30 consecutive potentially eligible septic shock patients admitted from June 2013 to November 2013, 5 patients were excluded from the study for calibration problems (3 because the VTIao was not correctly aligned and 2 because pulse wave transit time was not obtained). A total of 25 patients were included in the final analysis. Average age was 64 ± 15 years, most were men (Table 1). Average SAPS II and SOFA scores were 55 ± 21.3 and 13 ± 2, respectively. The most common origin of sepsis was community-acquired pneumonia (60%). ICU mortality was 36%.

**Table 1:** Baseline characteristics of the study population.

Variable	Overall (N = 25)	Responders (n = 12)	Non-Responders (n = 13)
Age, years	64 ± 15	60.3±15	67.4±14
Male, n (%)	15 (60)	6 (50%)	9 (69%)
SAPS II	55 ± 21	51±17	58±24
SOFA	13 ± 2	12±3	14±2
Comorbidities			
Diabetes mellitus	5 (20)	3 (25)	2 (15)
COPD	11 (44)	6 (50)	5 (38)
Heart failure	5 (20)	2 (17)	3 (23)
Origin of sepsis n (%)			
Community-acquired pneumonia	15 (60)	8 (66)	7 (54)
Abdominal infection	9 (36)	4 (33)	5 (38)
Cutaneous	1(4)	0	1 (8)
Origin of emergency admission, n(%)			
Emergency surgery	7 (28)	3 (25)	4 (31)
Medical	18 (72)	9 (75)	9 (69)
ICU mortality, n (%)	9 (36)	6 (50)	3 (23)
LVEF	54±9	51±10	56±8

Quantitative variables are expressed as mean±SD.

SAPSII, Simplified Acute Physiological Score II; SOFA, Sepsis-related Organ Failure Assessment; COPD, chronic obstructive pulmonary disease; ICU, Intensive Care Unit; LVEF, left ventricular ejection fraction.

### Comparison between CO_esCCO_ and CO_TTE_

Mean cardiac output at T0 was 5.8±1.35 L/min by esCCO and 5.27±1.17 L/min as assessed by CO_TTE_. At T1, the respective values were 6.63 ± 1.57 L/min for esCCO and 6.10±1.29 L/min for CO_TTE_.

**Fig 1:**
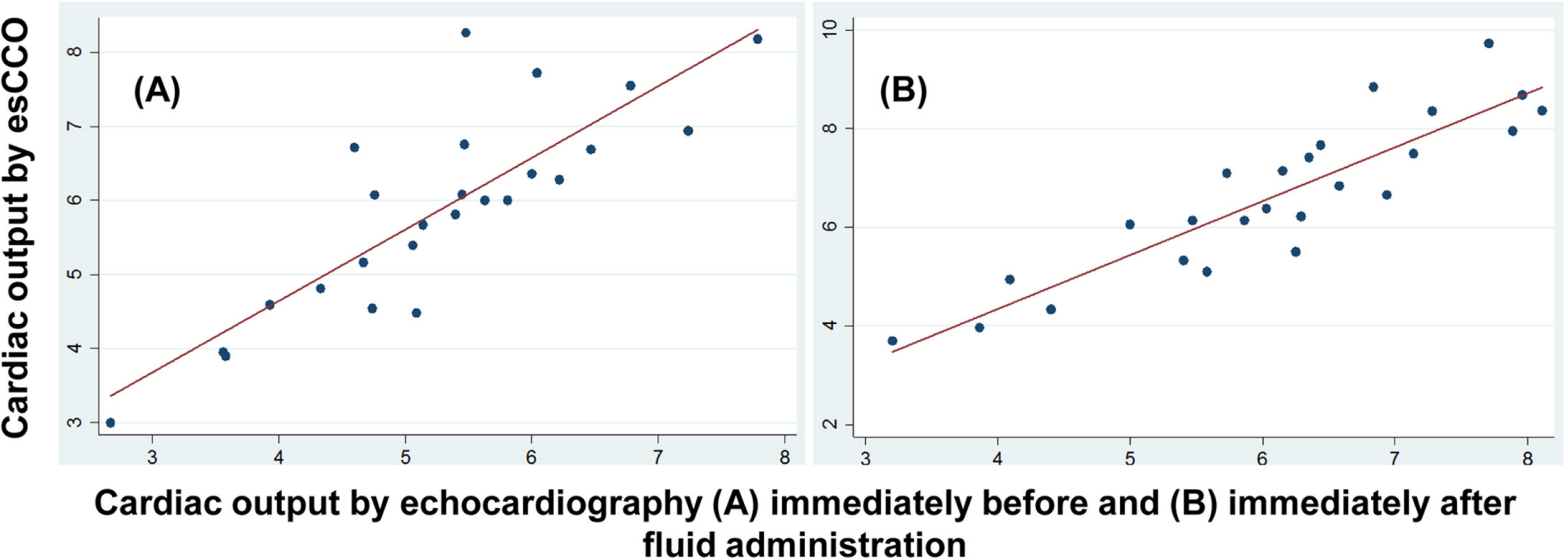
Linear correlation between cardiac output measured by the estimated Continuous Cardiac Output system (CO_esCCO_) vs measured by transthoracic echocardiography (CO_TTE_). (A) Immediately **before** administration of 500 mL of crystalloid solution over a 20-minute period (T0). (B) Immediately **after** administration of 500 mL of crystalloid solution over a 20-minute period (T1).

Fig 1A and 1B show the linear correlations between CO_esCCO_ and CO_TTE_ at T0 and T1, respectively r^2^ = 0.71 p < 0.0001 and r^2^ = 0.81 p < 0.0001.

**Fig 2:**
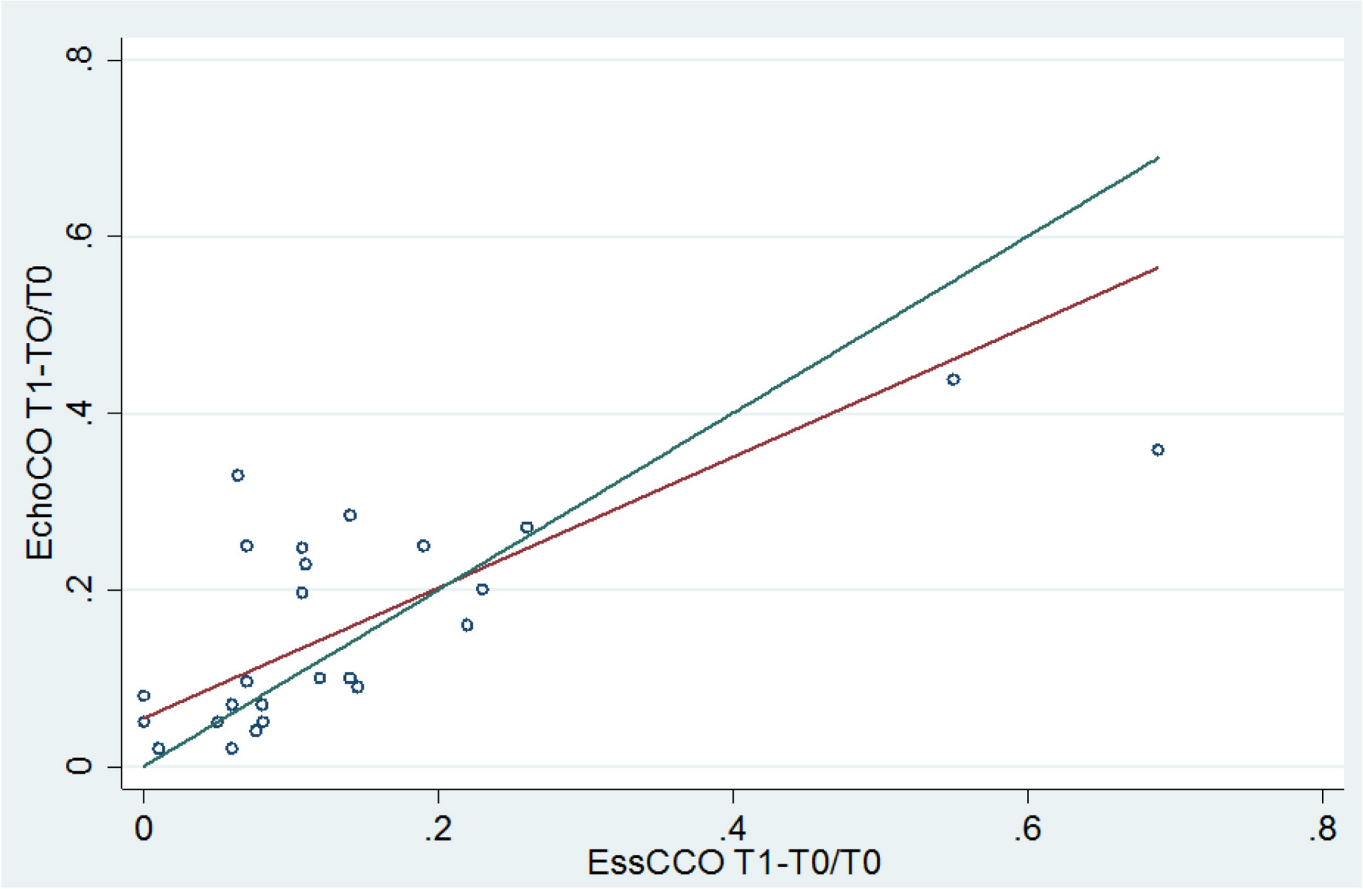
Linear correlation between change in estimated Continuous Cardiac Output (CO_esCCO_) and cardiac output measured by transthoracic echocardiography (CO_TTE_) between T0 (before fluids) and T1 (after fluids). The blue line represents the line of perfect concordance; the brown line is the reduced major axis, representing the line of best fit.

Fig 2 shows the linear correlation between CO_esCCO_ and CO_TTE_ change (from T0 to T1), r^2^ = 0.70 p < 0.0001.

The bias and limits of agreement (95% confidence interval) between CO_esCCO_ and CO_TTE_ at T0 and T1 were respectively -0.60 L/min (-2.05 to 0.85 L/min) and -0.54 L/min (-1.92 to 0.84 l/min.)

**Fig 3:**
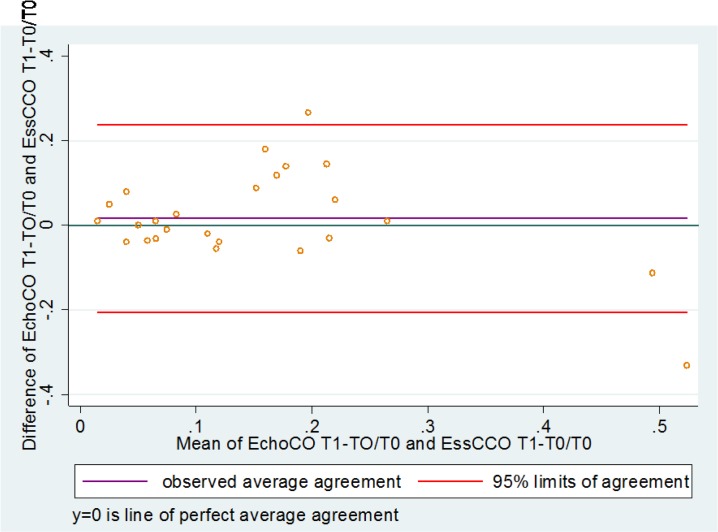
Bland and Altman plots for change in estimated Continuous Cardiac Output (CO_esCCO_) and cardiac output measured by transthoracic echocardiography (CO_TTE_) between T0 (before fluids) and T1 (after fluids).

The average bias of agreement (95% confidence interval) between T0 and T1 measured by CO_esCCO_ and CO_TTE_ was 0.2 L/min (-2 to + 2.4 L/min) (Fig 3).

Overall, 12 patients were classified as responders, and 13 patients were classified as non-responders by the reference echocardiography method. There was no significant difference in terms of norepinephrine dose between responders and non-responders at baseline (0.8±0.3 vs 0.7±0.4 gamma/kg/min respectively).

**Fig 4:**
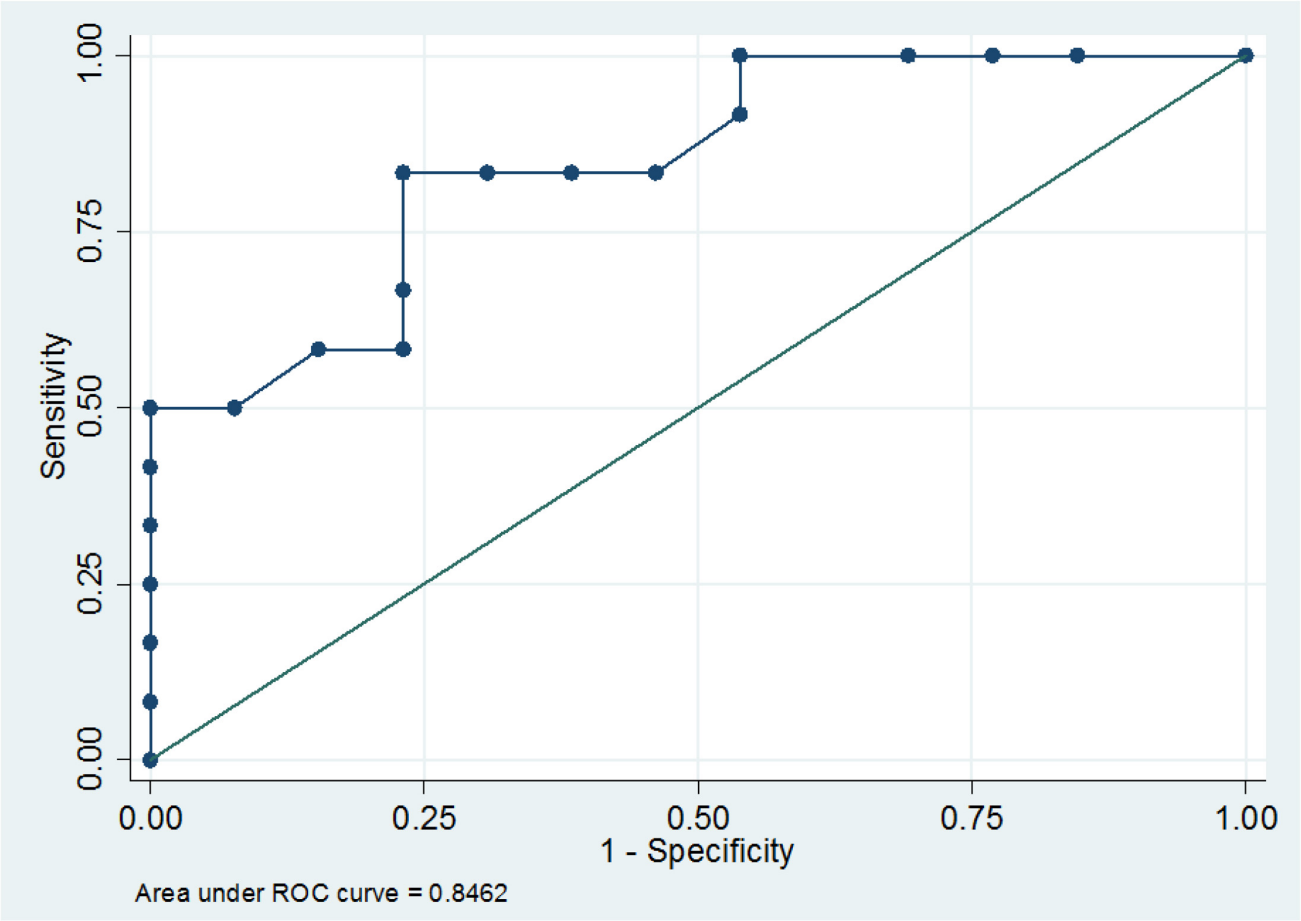
Receiver operating characteristic curve for change in estimated Continuous Cardiac Output (CO_esCCO_) between T0 (before fluids) and T1 (after fluids).

A threshold value of 11% increase in CO_esCCO_ was found to discriminate responders from non-responders with a sensitivity of 83% (95% CI, 0.52–0.98) and a specificity of 77% (95% CI, 0.46–0.95). The area under the ROC curve was 0.84 (95% CI, 0.69–0.99). The positive LR was 3.6 and the negative LR was 0.21 (Fig 4).

Intra-observer variability with CO_TTE_ was 7±4% and the inter-observer variability (MF and JB) was 7±5%.

## Discussion

The main finding of this study is that esCCO can detect rapid changes in CO in adult patients in the early phase of septic shock. The changes in CO determined by the non-invasive esCCO device were correlated with CO as measured by the reference method, namely transthoracic echocardiography (CO_TTE_) (r² = 0.70) after rapid fluid infusion. We also found that a threshold value of 11% increase in CO_esCCO_ after fluid infusion adequately discriminated responders from non-responders with high sensitivity (83%) and specificity (77%).

Recent studies compared CO measured by esCCO (CO_esCCO_) and by thermodidution [11, 25]. The authors reported a good correlation between the two methods, with a small bias (0.04 to 0.13 L/min) and limits of agreement -1.15 and 1.61 L/min. Comparison of esCCO vs CO measured by transthoracic echocardiography (CO_TTE_) has yielded discordant results. Mansencal et al[26] demonstrated the best correlation (R^2^ = 0.92) in a patients hospitalized in coronary care unit, with limits of agreement from -0.60 to +0.68 L/min. Bataille et al[27] also reported a good level of correlation between CO_esCCO_ and CO_TTE_ (R^2^ = 0.61) in a patients hospitalized in the Intensive Care Unit (ICU), but with a bias of -1.6 L/min and limits of agreement from -4.7 to + 1.5 L/min.

Appropriate early management of patients with septic shock is vital in view of the high mortality that persists in this patient population[2, 28–29]. The objectives of therapy in hemodynamic terms over the first 6 hours were recently underlined in an international consensus conference[30], namely to obtain central venous pressure of 8–12 mmHg, mean arterial pressure ≥ 65 mmHg, urinary output ≥ 0.5 mL/kg/h and superior vena cava oxygenation saturation (Scvo_2_) or mixed venous oxygen saturation (Svo_2_) of 70% or 65%, respectively. Measurement of central venous pressure or mixed venous oxygen saturation requires insertion of a central catheter (either by the jugular or subclavian route), which may not always be possible in critical care patients, particularly during the initial acute management. Similarly, the presence of a central line does not allow evaluation of cardiac output or response to vascular filling, once hemodynamic objectives have been achieved[9]. For these reasons, several indices have been developed in recent years to predict the response to fluid challenge, both static and dynamic measures that are based on the variations in pulsed pressure during mechanical ventilation. These indices are much more reliable, but require insertion of an arterial catheter, and measures should be performed in strict conformity with the conditions in which they were validated [18, 31]. In addition to these restrictions on the conditions for valid use, the presence of diastolic dysfunction can also invalidate these predictive indices [32]. Conversely, diastolic dysfunction does not affect the evaluation of cardiac output by Doppler, or the estimation of filling pressures.

Current trends in the management of critical care patients tend towards increasing use of non-invasive methods. For many years, there has been a focus on validating non-invasive techniques both to better understand pathophysiological mechanisms, and to improve management of hemodynamic instability[17, 33–34].

According to Vincent et al[6], a good hemodynamic monitoring system should provide accurate and reproducible measurements of relevant variables, provide interpretable data, be easy to use, readily available, and operator-independent, have a rapid response time, cause no harm, be cost-effective and provide information that can be used to guide therapy. The esCCO technique appears to fulfil the majority of these criteria, although the paucity of literature data precludes drawing any firm conclusions.

In our study, the response to fluid challenge was assessed using VTIao, which has previously been shown to be a valid indicator in septic shock patients[34]. VTIao is easy to measure, reproducible, and widely used in clinical practice. TTE is accepted as a reliable method for CO measurement in clinical practice and has been validated in comparison with thermodilution by several studies[13–14, 35–36].

In their study published in 2012, Bataille et al[27] reported a correlation coefficient of 0.61 between CO_TTE_ and CO_esCCO_ (p <0.001). The Bland and Altman analysis corrected for repeated measures showed a bias of -1.6 L/min, with limits of agreement from -4.7 to + 1.5 L/min, and a percentage error (2 SD/mean CO) of 49%. The correlation for CO changes was significant (R = 0.63, p< 0.001). In our study, the corresponding results were slightly better in terms of bias and limits of agreement, respectively -0.60 L/min (-2.05 to 0.85 L/min) at T0 and -0.54 L/min (-1.92 to 0.84 L/min.) at T1. The average bias of agreement (95% CI) between T0 and T1 measured by CO_esCCO_ and CO_TTE_ was 0.2 L/min (-2 to + 2.4 L/min). Several potential criticisms of the study by Bataille et al have been put forward[37], including the assertion that the authors did not standardize the timing of CO measurement, the clinical conditions and the reasons why the operator chose to measure CO. The study was also reproached for not having given any information about the physician who performed TTE or any information about patient characteristics. In designing our own study, we took these remarks into account to avoid similar pitfalls, and included a homogeneous population of patients, with a detailed protocol developed by a team with long experience in this area[17, 33–34], aided by an expert statistician. In the context of coronary care, Mansencal et al[26] observed similar results to those of our study, with a correlation coefficient between CO_TTE_ and CO_esCCO_ of 0.92 (p <0.001) in their study. Furthermore, they reported excellent agreement between CO as determined by esCCO and echocardiography, with a 95% confidence interval of -0.60 to +0.60 L/min.

In a meta-analysis of studies comparing techniques of cardiac output measurement using bias and precision statistics, Critchley et al[38] concluded that acceptance of a new technique should rely on limits of agreement of up to 30%. Other studies have been performed using other thermodilution techniques, such as intermittent bolus thermodilution CO measurement [11, 25], pulse contour CO measurements using the FloTrac system (Edwards Lifesciences)[39] or the PiCCO pulse contour system (Pulsion Medical systems)[40], with reportedly good correlation and good accuracy between these techniques[41].

In routine practice, the most important element is the system’s ability to record variations occurring in line with the patient’s hemodynamic condition, or during vascular filling. In our study, the average bias of agreement (95%CI) between T0 and T1 (after fluid infusion) as measured by CO_esCCO_ and CO_TTE_ was 0.2 L/min (-2 to + 2.4 L/min), in line with the findings of Bataille et al[27] and Mansencal et al[26].

Norepinephrine is a powerful vasoconstrictor that is frequently used to restore arterial pressure in circulatory failure. However, it is known to increase cardiac preload and cardiac index and reduce cardiac preload dependency, as recently shown by Monnet et al[31]. In our study, all patients were receiving norepinephrine at inclusion, although there was no significant difference between responders and non-responders in the dose. Nonetheless, we cannot exclude the possibility that high doses of norepinephrine in some patients may have masked cardiac preload dependency.

We believe that our study findings yield several arguments in support of the use of esCCO. Firstly, esCCO is a simple, non-invasive technique to evaluate hemodynamic status. Secondly, training ICU physicians in the use and interpretation of esCCO is quick, easy, and inexpensive. Thirdly, removing the need for central and arterial catheter placement represents a considerable time gain and avoids an additional puncture site that could be a potential port of entry for bacteria. Lastly, optimizing hemodynamic status as early as possible could help avoid progression to multiorgan failure, thereby reducing morbidity and mortality in patients with severe sepsis and septic shock.

However, some limitations of this work should be acknowledged. Firstly, this is a pilot study with a relatively small sample size, conducted in septic patients. This may explain the poor lower range of the CIs for sensitivity and specificity for distinguishing between responders and non-responders. Furthermore, although the use of esCCO has been validated in other contexts, this is the first study to examine the use of this technique in septic shock patients in the ICU, and therefore, no external validation in a larger population could be performed. Secondly, 2 patients were excluded because no esCCO value could be displayed. This is likely because it is difficult to obtain a reliable signal in patients who remain unstable with cold extremities. Thirdly, the patients must be have regular cardiac rhythm, which may limit the applicability of the device. However, our findings offer a proof of concept and suggest that further research would be appropriate.

In conclusion, this study shows a strong correlation between the two methods of measuring CO, namely esCCO and echocardiography, and the changes in CO following fluid infusion in ICU patients, with clinically acceptable limits of agreement. Therefore, we purport that the esCCO monitor can be recommended for critically ill patients.
